# Perceived facilitators and barriers in diabetes care: a qualitative study among health care professionals in the Netherlands

**DOI:** 10.1186/1471-2296-14-114

**Published:** 2013-08-10

**Authors:** Lieke GM Raaijmakers, Femke JM Hamers, Marloes K Martens, Charlotte Bagchus, Nanne K de Vries, Stef PJ Kremers

**Affiliations:** 1 Department of Health Promotion, NUTRIM School for Nutrition, Toxicology and Metabolism, Maastricht University Medical Centre, P.O. Box 616, 6200 MD Maastricht, The Netherlands; 2 ResCon, Research & Consultancy, Haarlem, The Netherlands; 3 Athena Institute, VU University Amsterdam, Amsterdam, The Netherlands; 4 Caphri, School for Primary Care and Public Health, Maastricht University Medical Centre, Maastricht, The Netherlands

**Keywords:** Diabetes, Health care professionals, Facilitators, Barriers

## Abstract

**Background:**

The need to understand barriers to the implementation of health care innovations in daily practice has been widely documented, but perceived facilitators and barriers in diabetes care by Dutch health care professionals remain unknown. The aim of this study was to investigate these factors among health care professionals (HCPs) using a qualitative research design.

**Methods:**

Data were collected from 18 semi-structured interviews with HCPs from all professions relevant to diabetes care. The interviews were recorded and transcribed verbatim and the data were analyzed using NVivo 8.0.

**Results:**

Major facilitators were the more prominent role of the practice nurses and diabetes nurses in diabetes care, benchmarking, the Care Standard (CS) of the Netherlands Diabetes federation and multidisciplinary collaboration, although collaboration with certain professional groups (i.e. dieticians, physical therapists and pharmacists), as well as the collaboration between primary and secondary care, could still be improved. The bundled payment system for the funding of diabetes care and the role of the health insurers were perceived as major barriers within the health care system. Other important barriers were reported to be the lack of motivation among patients and the lack of awareness of lifestyle programs and prevention initiatives for diabetes patients among professionals.

**Conclusions:**

Organizational changes in diabetes care, as a result of the increased attention given to management continuity of care, have led to an increased need for multidisciplinary collaboration within and between health care sectors (e.g. public health, primary care and secondary care). To date, daily routines for shared care are still sub-optimal and improvements in facilities, such as registration systems, should be implemented to further optimize communication and exchange of information.

## Background

Diabetes mellitus is a rapidly growing health problem, which affects approximately 366 million people worldwide [1]. The prevalence of diabetes in the Netherlands in 2011 was 801.000 and this number increased by 87,000 patients each year, thereby approaching 1 million (6% of the total population) [2]. The incidence concerned 45,000 men and 42,000 women (5,5 per 1.000 men and 4,9 per 1.000 women). In addition to these diagnosed patients, registration data in general practice showed that an estimated 25% of the patients is as yet undiagnosed in the Netherlands [3]. Reasons for this number of undiagnosed patients are amongst others the lack of attention of professionals for early symptoms of diabetes [4]. Furthermore, data on the prevalence of diabetes in nursing homes and on the prevalence of diabetes type 2 among children and adolescents appears to be lacking [4].

Diabetes is a complex and systemic chronic illness, which affects various organs and systems and is often accompanied by other diseases. Hence, diabetes requires continuous medical care and ongoing patient self-management and support to prevent acute complications, like hyperglycemia or hypoglycemia and reduce the risk of complications in the long run such as hypertension, cardiovascular diseases or kidney failure [5,6]. Continuity of care is concerned with the quality of health care for patients with chronic conditions like diabetes [6]. Management continuity is particularly important in chronic diseases such as diabetes, since the care for these patients requires optimal coordination and communication between the different health care professionals and organizations that contribute to the patients’ care [7-9]. Management continuity can be achieved when services are seamlessly linked and this is facilitated by shared management plans or care protocols [8]. Many aspects of the care for diabetes patients are nowadays managed by patients themselves on a life-long basis [10].

**Figure 1:**
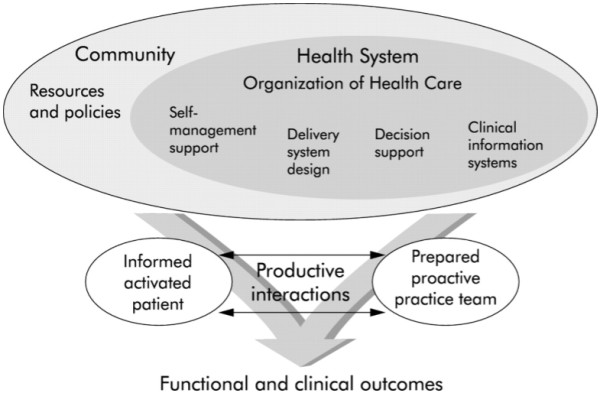
The chronic care model.

In recent years, multiple changes in diabetes care have been introduced in the Netherlands, with the aim of improving the continuity and quality of care. Attention to continuity of care has increased as a result of the 2008 initiative of the Dutch Ministry of Health, Welfare and Sports to start an integrated, programmatic approach to chronic diseases [11,12]. The concept of continuity of care is also reflected in the Chronic Care Model (CCM) (Figure 1), a framework that can be used to optimize the provision of care for patients with chronic conditions [13], and that advocates integrated care and disease management and the use of evidence-based care standards and guidelines [14]. The CCM focuses on improving and optimizing six key elements of the health care system: community resources and policies, organization of health care, self-management support, delivery system design, decision support and clinical information systems [15]. In Dutch diabetes care, the CCM is reflected in the National Diabetes Action Program (NAD), which is funded by the Ministry of Health, Welfare and Sports [16]. The overall purpose of the NAD (2009–2013) is to create the circumstances, conditions and instruments necessary to slow down the increase in the number of people with diabetes and to reduce complications in diabetes patients [16].

The main objective of the action program is the systematic implementation of the Netherlands Diabetes Federation (NDF) Care Standard (CS) for the content, organization, quality and funding of diabetes prevention and care [16]. A care standard is a general framework outlining the treatment of people with a specific condition, while clinical guidelines describe the content of care in more detail [17,18]. The NDF CS for type II diabetes mellitus describes the norm for generic multidisciplinary diabetes care and focuses on the content, organization and quality of diabetes care. The CS is constantly updated and extended, and is based on evidence-based guidelines [19,20]. It functions as a general overarching framework for the guidelines for the individual professional groups and focuses on a multidisciplinary approach to diabetes care. In addition, the CS is used as a purchasing instrument within the Dutch bundled payment approach. In the Netherlands, insurers purchase the services and care as described in the CS from a general contractor (called the Care Group)*,* which ends up in a so called bundled payment contract. Based on this contract, the Care Group assumes financial and clinical accountability and in turn subcontracts individual care providers (like the GP, dietician, internal specialist, etc.) or delivers parts of the services by itself [19]. Care standards are intended to provide health care professionals (HCPs), patients, researchers and funding bodies with a specification of the components of diabetes care, general treatment goals, and tools to evaluate the quality of care [5]. The Netherlands can be regarded as unique in the use of the CS for diabetes [17]. The NAD consists of five subthemes, which are in line with the concepts of the CCM and include activities to help achieve the main aim of the NAD: ‘Prevention’, ‘Position of the patient and client’, ‘Quality, organization and knowledge’, ‘Rules and funding’ and ‘E-communication and ICT facilities’. For each of these themes, it formulates instrumental objectives, which are implemented in various projects [16].

The introduction of innovations or changes in health care is widely recognized as a complex process with several factors affecting the process positively, i.e. good communication skills of professionals, or negatively, i.e. limited time and personnel resources [21]. Narrowing the gap between what we know and what HCPs actually do is a challenge that can help achieve effective and efficient health care [22]. The need to understand barriers to optimal health care and the dissemination and implementation of health care innovations in daily practice has been widely documented [22,23].

Previous studies have identified several of these facilitators of and barriers to effective care, operating at different levels in the health care system (the patient, the individual professional, the health care team, the organization of health care or the wider environment) [23,24]. Patient-related barriers include patient characteristics, such as lack of knowledge about diabetes [25,26], lack of motivation to change [26,27], low adherence [22,26], and a need for education [27]. Patient-related facilitators that have been found are early educational interventions at the start of the illness and patients’ ability to be responsible for and have control over their diabetes [25]. Examples of professional-related barriers are lack of motivation [28], lack of appropriate peer influence [22], lack of knowledge [22,26,28], not reading guidelines [29], lack of confidence in clinical skills [27,30], lack of effective communication tools, and lack of counseling and shared decision-making skills [30]. Professional-related facilitators include good communication skills that help to check patients’ needs and not overload them with information [25] and continuing medical education of physicians [27]. Barriers related to the health care team include suboptimal communication between HCPs [31,32], the lack of clear descriptions of professionals’ responsibilities within the team [26], and ignoring responsibilities of fellow professionals [25]. Additional barriers are the need for identical messages to the patients from all HCPs and competition between specialists and family physicans [26]. Working in a multidisciplinary team has been reported to be a facilitating factor associated with the health care team [25] as well as the beneficial effects of electronic data interchange on the frequency of communication between general practice and hospitals [33]. Examples of barriers in the organizational context are financial disincentives such as the lack of reimbursement [23,26,28,29]; organizational constraints such as the absence of organizational systems to support diabetes management (i.e. registries, automatic recall systems and reminder systems) [26]; and the lack of an individualized plan of care [34]. Organization-related facilitators are the importance of adhering to clinical practice guidelines and allocating time for patient education [27]. Examples of wider environmental characteristics that negatively influence guideline implementation in health care include limited time and personnel resources or services available for special populations such as the elderly and ethnic minorities [27,29] as well as work pressure [24,29,35,36]. Support from managers, including financial support to create opportunities to participate in educational meetings or to arrange the necessary materials or aids, and active involvement of superiors in the implementation process, have been reported as facilitators [37].

However, little is as yet known about the facilitators and barriers perceived by Dutch HCPs in diabetes care. This is of special interest since the Netherlands can be regarded as one of the frontrunners in implementing continuity of care and the CS. Therefore, the aim of this study was to investigate these facilitating and impeding factors among HCPs using a qualitative research design.

## Methods

### Participants

Data were collected from 18 semi-structured interviews with health care professionals held between November 2010 and January 2011. Participating professionals were selected based on their primary role in diabetes care, as described in the Care Standard [20]. Participants were randomly selected from a database of a previous quantitative study we conducted among health care professionals in the Netherlands [38]. In the questionnaire of that particular study participants were asked whether they were willing to participate in future research on the topic. The random selection of participants for our study resulted in a sample that was geographically dispersed in the Netherlands and professionals were not collaborating with each other. Consequently, selected participants were contacted by telephone and invited for participation and an appointment for an interview at their professional office was made. Participants included family physicians (FNs; n = 3), practice nurses (PNs; n = 3), diabetes nurses (DNs; n = 2) (one in primary care, one in secondary care), dieticians (DI; n = 3) (two in primary care, one in secondary care), physical therapists (PTs; n = 2) (primary care), internal medicine physicians (IPs; n = 3) and pharmacists (PAs; n = 2). The mean age of the participants was 43.9 years (range 31–59), 39% were male and 67% were working in primary care. Ethical approval for this study was not needed under Dutch law.

### Interview procedure

The interviews were semi-structured, with open-ended questions, and followed an interview guide based on the main theme of ‘Care Standard’ and the subthemes of the NAD. Each interview took place at the professional’s office and lasted approximately one hour. All interviews were audiotaped and transcribed by the interviewer (LR). The interview guide included the following themes: ‘background and function in diabetes care’, ‘general appreciation of diabetes care’, ‘organization of diabetes care’, ‘continuity of care’, ‘funding of diabetes care and the role of the health insurer’, ‘the CS’, ‘working in accordance with the CS’, ‘individual care plan’, ‘multidisciplinary collaboration in diabetes care’, ‘standardized registration and exchange of information’, ‘prevention and lifestyle interventions’, ‘benchmarking and health care quality’, ‘barriers to diabetes care and to working in accordance with the CS’ and ‘facilitators of diabetes care and of working in accordance with the CS’. Some examples of questions were: ‘What do you think of the organization of diabetes care in the Netherlands?’, ‘What is the role of the health insurers in diabetes care?’ and ‘What do you think of the exchange of information and communication about patient care between health care professionals in your care team?’

### Research model

We used the Chronic Care Model (CCM) [12] (Figure 1) to classify the facilitators and barriers into the following key elements of the health care system: community resources and policies, the way health care is organized, self-management support, delivery system design, decision support and clinical information systems [15]. We added the category of HCP-related factors to the model. *Community resources* refer to the need among provider organizations for linkages with community-based resources, e.g. exercise programs and senior centers, in order to improve chronic care. The element of *organization of health care* is concerned with the structure, goals and values of a provider organization and its relationship with purchasers, insurers and other providers. *Self-management support* involves collaboratively helping patients and their family to manage their chronic condition by acquiring the right skills, providing self-management tools and routinely assessing problems and accomplishments. The *delivery system design* refers to the creation of practice teams with a clear vision on the planned management of chronic conditions within the structure of the medical practice. *Decision support* is concerned with the integration of evidence-based standards and guidelines in daily practice. *Clinical information systems* involve computerized information which helps care teams comply with practice guidelines and standards, provide feedback to physicians and serve as records to assist planning individual patient care and conducting population-based care [15]. Finally, *HCP-related factors* refer to factors concerned with the individual health care professional, such as their motivation, knowledge of and affinity with diabetes care.

### Data analysis

Data were analyzed using the NVivo qualitative research software package, version 8.0. Meaning units (words or sentences) were labeled with codes and the first researcher (LR) grouped these codes into categories and subcategories. The coding scheme was derived from the CCM.). The codes were checked by and discussed with an independent co-researcher (FH) and disagreements were solved in a consensus meeting. In case of unsolved disagreements between LR and FH, a third researcher (SK) was consulted for a final decision. In case data did not fit with the CCM, they were grouped in additional categories where necessary.

## Results

List of abbreviations for HCPs

FP: Family physician

PN: Practice nurse

DN: Diabetes nurse

DI: Dietician

PT: Physical therapist

IP: Internal medicine physician

PA: Pharmacist

### Community resources and policies

One of the facilitating factors perceived by several HCPs (PN1, DN1, PT1, IP1) in the community was the *increased attention to diabetes in health care and the media*. In addition, one respondent (DI1) stated that diabetes is a popular topic in scientific research as well. One respondent (DI1) thought it was an impediment that *diabetes is often seen as a disease that a lot of people suffer from and that it is therefore not taken seriously enough* (see Additional file 1: Table S1 for quotes).

The majority of the respondents reported not to be aware of lifestyle programs and prevention initiatives that they could refer their diabetes patients to. One respondent (PN2) reported *not to have a list of local exercise facilities* and another respondent (PT2) agreed that they *have to look for opportunities in the neighborhood themselves using their personal network*. However, two respondents (FP1, PN2) reported that many lifestyle programs were available, but the problem was that *patients often relapse soon when they have to maintain their new lifestyle after the lifestyle program has ended*. Another respondent (FP2) reported that their *care group had drawn up a list of local initiatives*.

### Organization of health care

In relation to continuity of care, the majority of the respondents mentioned *the role of the practice nurses and diabetes nurses* as a very important improvement in diabetes care *since they are up to date on the most recent developments, have more time and are trained to organize their care efficiently*. However, two HCPs (PN2, DN2) reported the possible *decreasing expertise of FPs* as a *negative side effect* of this substitution of care. Two respondents (PN1, PN3) mentioned that *many changes have occurred in diabetes care and that it takes time to get used to these changes*.

The introduction of the first CS in 2003 coincided with the development of the ‘bundled payment’ approach for integrated chronic care [19]. The majority of the interviewees perceived the bundled payment system as a barrier to diabetes care because *it is not suitable for chronic conditions; it leads to egoism and higher costs and makes care less transparent*. One respondent (PN2) perceived the bundled payment system as beneficial since *the funding system used to be very fragmented, while the overall picture is very clear nowadays.*

The majority of the respondents reported the funding of diabetes care and more specifically the role of the health insurers as an important barrier. Four respondents (PN2, DI1, IP1, PA1) mentioned that *the health insurers have a lot of influence and are dominant*, while three other respondents (FP1, FP2, IP2) stated that *the collaboration with health insurers is very inflexible*. One respondent (FP2) perceived the role of the health insurers also as a facilitating factor, since they have *influenced the development of multidisciplinary care groups in a very positive way*.

### Self-management support

Overall, the professionals perceived the lack of motivation among patients to be hampering the delivery of their care. Additionally, one respondent (PN1) reported that *patients are unaware of the importance of self-management in diabetes*, and another respondent (IP3) thought that *patients’ own sense of responsibility is disappointing*. However, one respondent (PN3) perceived *a tendency towards self-management among patients* and another respondent (PN2) mentioned education as an important tool to increase compliance by patients. Two respondents (FP2, PN1) also reported to have experienced specific problems with hard-to-reach groups such as low SES patients or patients from ethnic minorities.

Several respondents (FPs, DI, PT2) mentioned *the added value of the individual care plan to motivate patients* and *its contribution to self-management by patients*. However, some respondents (FP3, PN3, IP1, DI2) doubted whether such a plan is *suitable for the average diabetes patient, who is not as motivated, independent and able to manage their diabetes as is assumed in the care plan*.

### Delivery system design

The majority of the HCPs perceived multidisciplinary collaboration to be effectively organized both within primary care and between primary and secondary care, and one respondent (DN2) also perceived good collaboration within secondary care. Several respondents (FP1, PN, DI3, PT1, PA2) perceived the *direct communication lines and short (physical) distances to other professionals* as facilitating the achievement of multidisciplinary collaboration in diabetes care. Furthermore, one respondent (PA) mentioned *systematic consultations with other professionals* as beneficial to collaboration.

The respondents also perceived barriers in relation to multidisciplinary collaboration. Some respondents would like to collaborate more with specific professional groups such as dieticians, physical therapists and pharmacists. One respondent (DN1) reported that *professionals in secondary care assume that they are collaborating better than professionals in primary care,* and that *health care needs to get rid of the island culture* in order to improve multidisciplinary collaboration. Two respondents (1, IP3) thought that *you need to have one or two leaders who maintain the collaboration* and *that systematic regional consultation* is desirable to improve the collaboration.

### Decision support

Overall, the interviewees perceived the CS to involve *all facets of diabetes care* and to *contribute to the quality of diabetes care*. The CS *is a solid agreement we made as professionals* (DN1); *it offers a clear framework* to check whether you have everything (IP3) and *provides unequivocal clarity* (DI3). However, one respondent (IP1) perceived *the lack of practice-based working* as a barrier, and two respondents (IP1, IP2) reported that *a risk of tunnel vision* as a result of the CS and its *sanctioning character* were barriers. One of the dieticians (DI2) mentioned that the CS describes everything comprehensively, except the part in which the dietician is involved. One of the internal medicine physicians (IP1) reported that *the use of protocols in care has led to the provision of care in accordance with agreements, but policy makers think that this automatically implies high quality care, which is a mistake*.

With regard to the implementation of the CS, one respondent (IP1) reported that the CS is either *insufficiently promoted or is known as a forcing model*. Another respondent (FP2) reported the need for *time to adapt to working in accordance with the current version of the CS*, before a new update is introduced.

### Clinical information systems

Overall, HCPs perceived the availability and use of a large number of different registration systems as a major barrier in terms of registration and exchange of information in diabetes care. These systems *are used by the so called Care Groups, practices and hospitals in the Netherlands to register patient information and health care quality indicators. HCPs perceived these systems* often as incompatible, which impedes communication. One respondent (PN3) indicated the use of the *same registration system by all professionals involved*, as well as the use of digital patient records as facilitating factors in improving the quality of care.

The majority of the respondents perceived the use of the principles of benchmarking as a positive development in diabetes care. Benchmarking was reported as a positive *feedback mechanism and stimulating factor* (FP3), *it makes professionals more aware* (DI1) and it is *helpful in the communication with health care insurers* (IP2). On the other hand, three respondents (FPs, PT2, PA2) perceived disadvantages of external benchmarking (by health insurers). In addition, the majority of the respondents reported that the quality of the indicators used for benchmarking acted as a barrier. According to the respondents, several indicators are *nonsensical or manipulated*, and *certain aspects of their care are not covered by the indicators*. Moreover, two respondents (IP2, PN3) mentioned that health insurers are interested in other indicators than those that are regarded as most important by the professionals. One respondent (IP1) argued that *the use of the current indicators and benchmarking principles leads to manipulation of information*.

### HCP-related factors

Barriers and facilitating factors with regard to HCPs were related to their training, professional vision, image and affinity with diabetes care. Two respondents (PN3, DN1) reported *the high educational and knowledge levels of Dutch health care professionals* as a facilitating factor. However, two participants (PN2, IP3) perceived the *lack of expertise among FPs* as an impediment, and another HCP (IP1) reported that colleagues *still use unqualified staff for the tasks of practice nurses and diabetes nurses*.

The respondents perceived barriers in relation to the role and image of dieticians and physical therapists. Both of the dieticians working in primary care whom we interviewed felt that they had a negative reputation among patients. Dieticians *used to be seen as people who provided rules about what people were allowed and especially not allowed to eat, and this image still prevails among patients, making them unwilling to consult dieticians*. One of the participating FPs (FP2) confirmed that this reputation also existed among professionals. Several respondents took the view *that physical therapists need to be aware of their role in diabetes care.* One of the participating physical therapists (PT1) held the opinion that the role of their profession in diabetes care is too limited, and emphasized that their role is mainly concerned with eliciting behavioral change in patients.

Two respondents (PN1, DN2) reported that the *lack of affinity with diabetes* in HCPs was a barrier. In Dutch health care, family physicians are automatically involved in the care for patients with type 2 diabetes and a high risk of diabetes. These participants argued that FPs do not always have affinity with diabetes care because of their more general function and therefore diabetes care should preferably be provided by FPs with an additional education in diabetes, which is available to in the Netherlands.

## Discussion

This study has contributed to our understanding of facilitators and barriers perceived by Dutch health care professionals in diabetes care. Using the CCM to identify such barriers and facilitators from the perspective of health care professionals resulted in a structured overview of these factors. One major facilitator we found was the more prominent role that practice nurses and diabetes nurses in diabetes care have been given since the introduction of continuity of care and more specifically management continuity in the Netherlands, and which is greater than in other health care domains. These nurses can play an important role in educating patients and encouraging adherence; and in certain situations they can even replace physicians in delivering many components of diabetes care [39]. Moreover, a recent study of Dutch diabetes care found that standardized diabetes care, delivered by a nurse specialized in diabetes, is a good alternative to standard care by an internal medicine physician, with comparable results after one year in terms of treatment goals, and even better results in terms of patient goals and cost-effectiveness [40]. In contrast, the role and image of dieticians and physical therapists were reported as barriers. Dieticians have (or perceive themselves as having) a negative reputation among patients and fellow professionals. A previous Dutch study also reported the image of the dietician to be a barrier to the collaboration with family physicians [41]. The role of physical therapists in diabetes care seems somewhat unclear and too limited in the opinion of the physical therapists themselves.

Another major facilitator is multidisciplinary collaboration, although the collaboration with certain professional groups (i.e. dieticians, physical therapists and pharmacists) could be further improved, as could the collaboration between primary and secondary care. In line with our results, a previous study among Belgian family physicians reported the competition between specialists and family physicians to be a barrier to evidence-based diabetes care [26]. Our respondents also perceived the large number of different registration systems to record and exchange information in diabetes care as a major barrier to collaboration and communication. Previous studies reported suboptimal communication between HCPs to be a major problem in relation to shared care [31,32] and the beneficial effects of electronic data interchange on the frequency of communication between general practice and hospitals [33].

Our respondents perceived the CS as a facilitator, since they perceived it to be a clear framework that contributes to quality of care. This is in agreement with the findings of studies on clinicians’ attitudes towards guidelines, which showed that clinicians agree that guidelines contribute to the quality of care [42].

Benchmarking was also perceived as a major facilitating factor. Previous studies have concluded that benchmarking is a promising tool for quality improvement in chronic care in general, and in diabetes care specifically, but until now there has been a striking lack of clinical evidence from controlled trials [43]. However, the majority of our interviewees reported the quality of the indicators for benchmarking to be a barrier. A recent report on the development of European quality indicators for primary diabetes prevention programs stated that even though it is not possible to develop an error-free measure of quality, an indicator should always be tested for feasibility, reliability and validity during its development phase. Furthermore, professionals and health care insurers should always keep in mind that indicators just indicate and that they probably never completely capture the quality of the health care system. Even the best indicators have limitations, and these limitations should be taken into consideration when drawing conclusions based on the indicators [44].

The bundled payment system for the funding of diabetes care and the role of the health insurers were perceived as major barriers within the health care system. Our respondents thought that the bundled payment system is unsuitable for chronic conditions and is counterproductive. However, early results from the adoption of bundled payment for diabetes care in the Netherlands show that it has improved the organization and coordination of care and has led to better collaboration between health care professionals and better adherence to care protocols [45]. Moreover, many services and health care providers seem positive about the organizational improvements in care resulting from the introduction of the bundled payment approach [46].

Another important barrier perceived by our respondents was the lack of motivation on the part of patients, which is consistent with the findings of previous studies reporting a lack of motivation to change among patients [26,27]. By contrast, our respondents perceived the use of individual care plans to be a facilitator contributing to self-management by patients. A previous study among Canadian physicians identified the individual care plan as a tool to achieve improved communication in the transition from specialist to primary diabetes care [47]. Despite the reported added value of individual care plans, our respondents doubted whether such plans are suitable for the average diabetes patient, who is not as motivated, independent and able to manage their diabetes as assumed in the care plan. This finding is similar to the results of a study among Belgian family physicians, who questioned the feasibility and desirability of implementing clinical guidelines in an older diabetic population [26].

The main barrier in relation to community resources was reported to be the lack of awareness of lifestyle programs and prevention initiatives for diabetes patients among professionals. Programs meeting requirements for cost-effective lifestyle interventions have been developed in the Netherlands and have been implemented in several pilot projects in primary care, examples being ‘Beweegkuur’ (Exercise therapy) [48] and ‘Exercise on prescription’ [49]. The familiarity with such programs, as well as access to them, should obviously be improved. In an attempt to promote quality assurance and control, a registration and assessment system for health education and health promotion interventions has been developed in the Netherlands [50]. Whether this system will help ensure that the most effective and efficient interventions are implemented and disseminated can as yet not be guaranteed [50], but at least a comprehensive national list is being put together.

Some strengths and limitations of the current study remain to be addressed. A strength of this study is the use of semi-structured interviews which provide the opportunity to collect in-depth information and understand understand perspectives and experiences of participants. Moreover, by using the CCM to categorize the identified facilitators and barriers, we increased the standardization of reporting facilitators and barriers in relation to diabetes care. Our results show that expansion toward better integration of prevention and health promotion in the CCM would be useful [51]. Furthermore, all interviews were conducted by the same researcher, in order to increase consistency in the data collection process, and all codes were checked independently by two researchers to increase conformability (objectivity and neutrality).

A limitation of the current study is the selectivity of the sample; since participation was voluntary and recruitment was conducted through a random selection of professionals from a database of a previous study on the same topic, it is plausible that the current sample had greater affinity with and involvement in diabetes care than the population of all Dutch care providers, which may limit the generalizability of our findings. Furthermore, we selected a heterogeneous sample of health care professionals because of their primary role in diabetes care and in relation to the multidisciplinary approach in Dutch diabetes care. However, the consequence of including this heterogeneous group was that we were only able to include 2 or 3 professionals per group and cannot guarantee that saturation has been achieved. Furthermore, we retrieved divergent views on some aspects of care and due to the relatively small sample size per professional group, we were not able to obtain consensus on all issues. Additionally, the current study is primarily focused on the organization of diabetes care and the use of the Care Standard in the Netherlands. Our results can however inform and support similar future approaches to organize diabetes care in other countries as well.

## Conclusion

The substitution of care originally provided by family physicians and specialists to practice nurses and diabetes nurses seems to work out well. These organizational changes in diabetes care, as a result of increased attention given to continuity of care, have led to an increased need for multidisciplinary collaboration within and between health care sectors (including public health, primary care and secondary care). A new CS could be helpful in this respect. To date, daily routines for shared care are still sub-optimal and facilities such as registration systems should be improved to further optimize communication and exchange of information.

## Competing interests

The authors declare that they have no competing interests.

## Authors’ contributions

LR was the primary researcher, collected all data and wrote the first draft of the manuscript. LR, MM, CB and SK were involved in determining the design of the study. LR and SK were involved in the decisions on the analyses. LR and FH were involved in transcribing and coding the interviews. SK was consulted in case of inconsistent coding. All authors were involved in revising the manuscript and have read and approved the final version of the manuscript.

## Pre-publication history

The pre-publication history for this paper can be accessed here:

http://www.biomedcentral.com/1471-2296/14/114/prepub

## Supplementary Material

Additional file 1: Table S1 Perceived facilitators and barriers in diabetes care. Click here for file
