# Supplementary material for: Perceived facilitators and barriers in diabetes care: a qualitative study among health care professionals in the Netherlands
Source: BMC Fam Pract. 2013 Aug 10;14:114. doi: 10.1186/1471-2296-14-114 (PMC3751909; doi:10.1186/1471-2296-14-114)
Supplement: Additional file 1: Table S1 — Perceived facilitators and barriers in diabetes care. [file 1471-2296-14-114-S1.doc]

**Additional file**

**Table 1: P**erceived facilitators and barriers in diabetes care

| **Category of factors** | **Quotations to illustrate the factors identified** |
| --- | --- |
| **Community resources and policies** | |
| *Attention* | (+) ‘Diabetes is receiving a lot of attention in primary and secondary care and also from politics, which has led to an improvement of the care’  (+) ‘There’s a lot of attention for diabetes in the media, which makes professionals and patients aware of the seriousness and that’s a good thing’  (+) ‘Diabetes is a popular topic in scientific research’  (-) ‘Diabetes is often seen as a common disease that a lot of people have and is therefore not taken seriously enough’ |
| *Prevention and lifestyle interventions* | (+) ‘We’ve made a list of local initiatives with our care group, so I know where to find them’  (+) ‘I received an overview from the municipal Sports Service listing facilities in the neighborhood, which is very convenient’  (-) ‘We don’t have a local list of exercise facilities’  (-) ‘There are so many programs for diabetes patients, but when the programs stop, the patients have to continue on their own and often they relapse soon’  (-) ‘It’s often the case that family physicians don’t know where to refer people for lifestyle changes’  (-) ‘You have to look for programs and possibilities in the neighborhood yourself, using your personal network’ |
| **Health care organization** | |
| *Continuity of care* | (+) ‘The accessibility of care in the Netherlands is very good’  (+) ‘Since practice nurses have been introduced in practices, everything has improved’  (+) ‘The best thing is that we can transfer care to the practice nurse’  (+) ‘We couldn’t do without the diabetes nurses’  (-) ‘A lot has changed in diabetes care over the past 10-15 years that you can’t keep up with’  (-) ‘We need to make sure that family physicians don’t lose their expertise in diabetes care because of the substitution of care by the practice and diabetes nurses’ |
| *Bundled payment system* | (+) ‘The funding used to be very much fragmented, while the entire picture is very clear nowadays’  (-) ‘An advantage of the bundled payment system is supposed to be that you’re working in closer collaboration with colleagues and paramedics, but that’s a farce of course’  (-) ‘Everyone is only fighting for themselves and that doesn’t make it cheaper’  (-) ‘Diabetes is not easy to cover in a bundled payment system, since the system was not developed for chronic conditions’  (-) ‘The bundled payment funding is very counterproductive’  (-) ‘The aim of the bundled payment system was to create transparency, but it’s become even more opaque’ |
| *Role of health insurers* | (+) ‘The health insurers do what they have to do and the prices are established by the Dutch Healthcare Authority (government institution)’  (+) ‘Health insurers have influenced the development of multidisciplinary care groups in a very positive way’  (-) ‘The health insurers are very restrictive in the services and medication they reimburse, which leads to a decrease in the quality of care delivered’  (-) ‘In our current health care system, the health insurer is dominant’  (-) ‘The collaboration with the health insurers is very inflexible’  (-) ‘The conversations with the health insurer take a lot of time and effort because of the competing interests’  (-) The health insurers have great power and that’s a problem because they think about money first and quality second’ |
| **Self-management support** | |
| *Motivation* | (+) ‘If you provide patients with enough education and you get to know the individual patient, their compliance greatly improves’  (+) ‘There is a tendency toward more self-management by patients and that’s very good’  (-) ‘The problem in health care is to motivate patients’  (-) ‘Patients’ own sense of responsibility is disappointing’  (-) ‘A lot of patients are fairly passive and dependent on medication’  (-) ‘The majority of the patients practice self-management, but some are still unaware of its importance in diabetes’ |
| *Individual care plan* | (+) ‘I think it’s a positive development that patients are guided towards self-management’  (+) ‘I absolutely see the added value of individual care plans to motivate patients’  (+) ‘If it works out I completely support the use of individual care plans since your patients become more motivated and take responsibility for their own health’  (-) ‘It’s good to make patients the masters of their own disease, but the problem is that the average type 2 diabetes patient is not the patient described in the care plan’  (-) ‘The problem is that we have a lot of different patient groups and it’s impossible to make different individual care plans for each group’  (-) ‘I think a lot of patients still adopt a dependent attitude toward their doctor and don’t want an individual care plan’ |
| *Difficult to reach groups* | (-) ‘We encounter specific problems because our practice is located in a low SES neighborhood’  (-) ‘Consultations that involve an interpreter take at least a third of the usual time extra’  (-)’The problem is to motivate female migrants to become physically active’ |
| **Delivery system design** | |
| *Multidisi-plinary collabo-ration* | (+) ‘In this region the collaboration between primary and secondary care is pretty well organized’  (+) ‘We work in a multidisciplinary team on the same floor, so we can easily ask each other things’  (+) ‘The short physical distances and direct communication lines to other professionals make it easy to have frequent contacts’  (+) ‘Systematic consultations with other professionals are beneficial for the collaboration’  (-) ‘There’s some arrogance among secondary care professionals because they assume they are collaborating better inside the hospital than we can in primary care’  (-) ‘We’ve been trying to get rid of the island culture for 10-15 years now’  (-) ‘The role of the dieticians should be bigger; they’re actually a very important factor’  (-) ‘There should be a whole team surrounding the patient, but that’s what I still feel is missing at the moment’  (-) ‘I think the collaboration between the family physicians and other primary care partners such as dieticians, physical therapists and pharmacists could be greatly improved’  (-) ‘You need one or two leaders who bring everyone together; when they withdraw, the continuity of care falls apart’ |
| **Decision support** | |
| *Care Standard* | (+) ‘I think the CS covers all facets of diabetes care’  (+) ‘The CS is a solid agreement we make as professionals’  (+) ‘It contributes to high quality care and provides a good basis for all disciplines involved’  (+) ‘It offers a clear framework and you can check whether you have everything’  (+) ‘The CS provides unequivocal clarity, so you know your colleague is doing the same’  (-) ‘In essence, the CS is fine, but I would suggest to work to some extent practice-based as well as evidence-based’  (-) ‘My professional group is afraid that the CS is going to dominate the care process and lead to tunnel vision and barriers’  (-) ‘The problem with the CS is that you’re not going to read it until you have patients to whom it applies’  (-) ‘A barrier could be that the CS is seen as sanctioning’  (-) ‘Everything is described in detail, except the part for the dietician’ |
| *Implemen-tation of CS* | (-) ‘I think it’s important to promote the CS as a framework to check whether your care is within the right parameters, but it shouldn’t be used as a forcing model’  (-) ‘Give professionals some time to adapt to working with the CS; the previous version was only 3 years old’  (-) ‘More attention for the CS from professional organizations would be useful’ |
| **Clinical information systems** | |
| *Bench-marking* | (+) ‘Transparency is very important and if you show this, health insurers notice it and act upon it’  (+) ‘I think the benchmark as a feedback mechanism and stimulating factor is good’  (+) ‘I think it can improve the care for patients since it makes you aware’  (+) ‘The principles of benchmarking are very good, but you need to make sure that you can use the information that professionals need to record anyway’  (+) ‘I think your professional group should take care of good quality, so the  internal benchmark is a good thing. External could be good, but you need to be careful since the information is easily misinterpreted’  (-) ‘Certain indicators, such as blood pressure, would always be good, but for other indicators you should be careful in interpreting them and they should ideally involve information like the neighborhood in which patients are living’  (-) ‘A lot of basic care is not covered by indicators’  (-) ‘The most important disadvantage is that they may be misused for financial settlements. That’s a bad development as regards transparency’  (-) ‘The information is used to manipulate in ways that are very counterproductive’  (-) ‘At the moment we use indicators that are nonsensical, and other indicators are manipulated’ |
| *Standar-dized registration and exchange of information* | (+) ‘We have a system in which you can record everything regarding the patient, and other professionals can see the information that’s relevant to them’  (+) ‘Working with the electronic patient record system has increased the quality of our care’  (-) ‘The systems used by different professionals are not compatible, so you can’t communicate through the systems’  (-) ‘After referral to secondary care there is stagnation of information provision, because of different registration systems’  (-) ‘At the moment there are 10-15 different systems in use’  (-) ‘Each hospital has its own system and the systems don’t correspond’  (-) ‘One uniform system for al family physicians would be perfect, but that’s an illusion’  (-) ‘The exchange of information between pharmacists and hospitals is fraught by lots of mistakes’ |
| **HCP-related factors** | |
| *Education* | (+) ‘I think all Dutch health care professionals are well-trained’  (+) ‘We should grade the knowledge level of professionals in diabetes care as 8 out of 10; it’s one of their strong points’  (-) ‘I see that family physicians make decisions based on insufficient knowledge’  (-) ‘Unfortunately the entire family physician training program includes only two days devoted to diabetes’ |
| *Image* | (+) ‘The dietician’s contribution is well acknowledged’  (+) ‘Dieticians have a very important role to play in diabetes care’  (-) ‘We family physicians agree that the reputation of dieticians needs to be improved, as the general view is that they only say what you’re not allowed to do’  (-) ‘Dieticians used to have a negative reputation, so patients still don’t want to be referred to them, while our daily practice has changed a lot’  (-) ‘Physical therapists need to be aware that they have a limited role in diabetes care because consulting a physical therapist is not necessary for all patients’  (-) ‘Physical therapists need to be aware that their role not only concerns getting patients to become physically active, but also eliciting behavioral change in patients’  (-) ‘The role we physical therapists are allocated in diabetes care is too limited’ |
| *Affinity* | (+) ‘At a health center you can distribute the fields of interest, and those professionals who have an affinity with diabetes care keep up to date in that field’  (-) ‘The lack of affinity with diabetes among professionals working in one-doctor practices has a negative influence on the quality of their care’  (-) ‘Family physicians have less affinity with patient self-management and education than the practice nurses’ |

(+) facilitators, (-) barriers
